# Flame Retardant Paraffin-Based Shape-Stabilized Phase Change Material via Expandable Graphite-Based Flame-Retardant Coating

**DOI:** 10.3390/molecules25102408

**Published:** 2020-05-21

**Authors:** Ling Xu, Xuan Liu, Rui Yang

**Affiliations:** Department of Chemical Engineering, Tsinghua University, Beijing 100084, China; xu-l16@mails.tsinghua.edu.cn (L.X.); liu-x15@mails.tsinghua.edu.cn (X.L.)

**Keywords:** shape-stabilized phase change material (SSPCM), flame-retardant property, surface coating, paraffin, expandable graphite (EG)

## Abstract

Shape-stabilized phase change material (SSPCM) is a promising thermal energy storage material in energy-saving buildings. However, its flammability leads to a fire risk. The conventional bulk addition method has a limited flame-retardant effect. Herein, a series of surface coatings with various flame retardants were introduced to improve flame retardance of SSPCM. The results showed that all of the coatings had flame-retardant effects on SSPCM; In particular, the EG coating performed the best: the horizontal burning time was the longest, the limiting oxygen index was above 30%, the V0 classification was obtained, the peak heat release rate was sharply decreased from 1137.0 to 392.5 kW/m^2^ and the burning process was prolonged with the least total smoke production. The flame-retardant mechanism was discussed. As paraffin easily evaporated from the SSPCM at a moderate temperature, it caused flames. After being surface coated, the EG-based coatings first hindered the volatilization of paraffin at a moderate temperature, then expanded and formed thick porous carbon layers at a high temperature to block the transfer of combustibles, oxygen and heat between the bulk and the environment. Therefore, the surface coating strategy achieved a desirable flame-retardant level with fewer flame retardants.

## 1. Introduction

Efficient energy-saving technologies are urgently required in buildings so as to address the massive energy consumption and greenhouse gas emissions due to rapid urbanization and modernization [1]. Introducing phase change materials into building envelops (walls, floor, roof, windows, etc.) is a passive and cost-effective technology to improve energy utilization efficiency [2,3,4]. Among them, paraffin-based shape-stabilized phase change material (SSPCM), which melt-blends paraffin with polymers, is flexibly processed and has no leakage problem during phase change, as paraffin is constrained in the polymer network [5]. It can store solar energy or waste heat as latent heat in order to control temperature fluctuation.

However, the application of SSPCM comes with a fire risk. Besides polymers, SSPCM also contains a large amount of paraffin in the matrix, which transforms into the liquid state above the phase change temperature. At high temperatures, traditional polymers first decompose into small molecules, which then gasify into the air and spark fires [6], whereas paraffin gasifies directly without decomposition and causes combustion at a relatively low temperature. The different flammability nature will determine a different flame-retardant mechanism for SSPCM.

Hence, far, the bulk addition method, introducing various flame retardants into SSPCM bulk, is the most widely used method to improve the flame retardance of SSPCM [7,8,9,10,11,12,13,14,15,16,17,18,19,20]. The typical flame retardants include the following:

(1) Inorganic fillers like Talc, CaCO_3_, antimony trioxide, organic montmorillonite (OMMT) and expandable graphite (EG). They act as physical barriers to lower the penetration of oxygen into the matrix and slow down the escape of combustibles from the matrix and thus improve the flame retardance of SSPCM. Wang et al. [7] added OMMT and EG into a high-density polyethylene (HDPE)/styrene-butadiene-styrene copolymer (SBS)/paraffin system and the flame retardance was enhanced. Once exposed to fire, the layer structure in OMMT and the pore structure in expanded graphite were both conducive to the trapping of gaseous paraffin in the matrix and slowed the spread of flames. In addition, OMMT and expanded graphite contributed to the formation of a char layer, which could hinder the exchange of heat and material between the matrix and the environment and thus delay the decomposition of SSPCM. Mochane et al. [8] studied the effect of EG and Cloisite 15A clay on the flame retardance of ethylene-vinyl acetate copolymer (EVA)/wax and the results showed that EG/Cloisite 15A clay was efficient, contributing to the dense char layer that resisted the release of flammable volatiles and the penetration of oxygen and heat.

(2) Reactive flame retardants like hydroxides (Mg(OH)_2_, Al(OH)_3_, etc.); red phosphorous (RP); metals (Fe, Mg, Al, Zn, etc.); and intumescent flame retardants (IFRs), such as ammonium polyphosphate (APP), pentaerythritol (PER), melamine (MA). They react at a certain temperature and promote the generation of a carbon char layer and inert gases. Reactive flame retardants can strengthen the char residue in the matrix and insulate the exchange of volatile products and heat, while the inert gases generated can dilute the oxygen and combustible gas concentration to some degree. All of these flame retardants contribute to the failure of the fire triangle and thus enhance the flame retardancy of SSPCM. Song et al. [11] explored the effect of nano-Mg(OH)_2_ and RP on an ethylene propylene diene monomer (EPDM)/paraffin system and confirmed that the flame retardance was better because of the MgO and H_2_O generated during burning. Zhang et al. [12] demonstrated that iron could facilitate the generation of ammonia and water from APP and MA, thus the oxygen concentration was decreased significantly. APP reacted with Fe^n+^ to strengthen the barrier effect of the char layer on combustibles and heat.

(3) Some flame-retardant compositions consist of inorganic fillers and reactive flame retardants and their synergistic effects further strengthen the flame retardance. Cai et al. [13] investigated the synergistic effect between EG and APP, helping to enhance the thermal stability and to suppress the flammability of HDPE/paraffin composites. This was ascribed to the formation of the heat-insulated phosphor-carbonaceous char residue from the reaction of APP and EG, accompanied by the capture of H• (or HO•) by PO• and from the dilution of gaseous combustibles by NH_3_ and H_2_O. In addition, EG itself also had the function of heat isolation. Sittisart et al. [9] proposed that formulations containing IFRs (e.g., APP/PER/montmorillonite (MMT) and APP/EG) were better candidates to improve the fire retardance of HDPE/paraffin, because of the synergistic effect between treated MMT (or EG) and IFRs, with a larger amount of residue and a thicker heat barrier.

Although the bulk addition method was demonstrated to be effective, the samples were still flammable during burning. In addition, the loadings of the flame retardants were always large (20–40 wt%). It would worsen the thermal storage capacity of SSPCM greatly, which is one of the most important performances of SSPCM.

In our previous work [21,22], we found that concentrating flame retardants on the surface of samples was effective to improve the flame retardance of SSPCM. In contrast to bulk addition, SSPCM with EG coating achieved good flame-retardant properties with fewer flame retardants.

Why did these different flame-retardant effects happen on SSPCM? Was it owing to the different flammability nature between SSPCM and traditional polymers? In this study, we investigated the effects of coatings containing various flame retardants such as Talc, OMMT, EG, APP, IFR (APP/PER/MA = 1/1/1), Al(OH)_3_ and RP. We found that all the coatings were able to improve the flame retardance of SSPCM in different degrees. In particular, EG-based coatings exhibited better flame-retardant effect with fewer flame retardants and V0 level was achieved at a certain loading. The flammability nature of SSPCM was clearly revealed and the flame-retardant mechanism of the surface coating strategy was proposed as well. This work will be helpful to choose appropriate flame retardants and prepare flame-retardant SSPCMs for industrial application.

## 2. Results and Discussion

### 2.1. Effect of Different Flame Retardants on SSPCM

**Figure 1:**
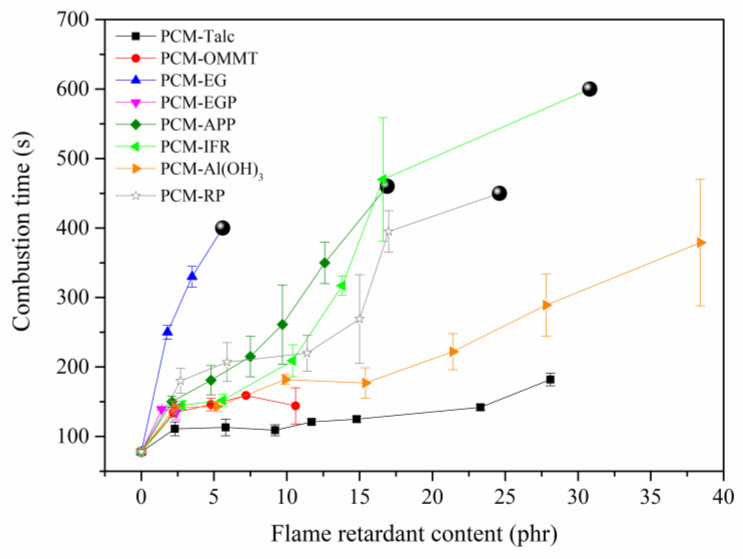
Horizontal burning results of paraffin-based shape-stabilized phase change materials (SSPCMs) coated with different flame retardants (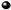: self-extinguish).

Flame retardants including inorganic fillers (Talc, OMMT, EG and expanded graphite plate (EGP)) and reactive flame retardants (APP, IFR, Al(OH)_3_ and RP) were introduced to study the flame-retardant effects on SSPCM. Figure 1 shows the horizontal burning results of SSPCMs. For PCM, the combustion time was less than 100 s. For PCM-Talc, PCM-OMMT and PCM-EGP, the combustion times were increased slightly because of the weak physical barrier effects of the three flame retardants. For PCM-APP, PCM-IFR and PCM-RP, the combustion times were greatly increased and self-extinguishing was observed. Those flame retardants reacted to form dense layers at a high temperature and provided better physical barrier effects than Talc, OMMT and EGP. For PCM-Al(OH)_3_, however, H_2_O was generated during burning and destroyed the protective layer, thus weakening the barrier effect to some extent. For PCM-EG, the combustion time was the longest and it self-extinguished with a much lower EG content.

**Figure 2:**
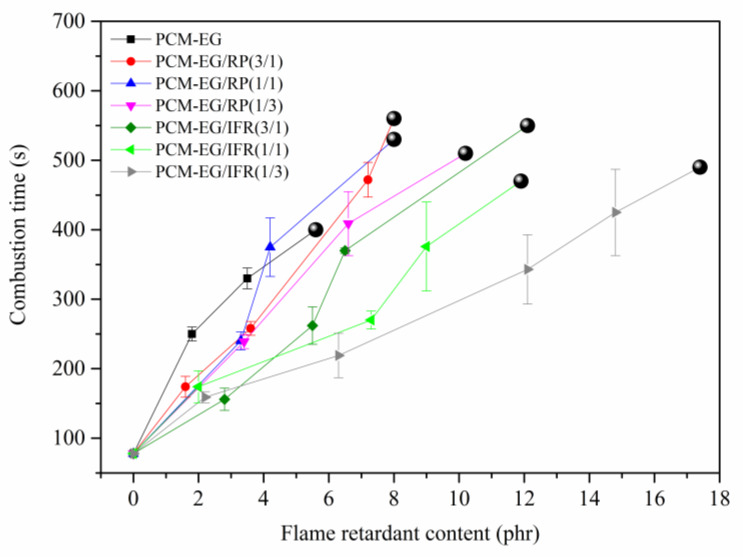
Horizontal burning results of SSPCMs coated with expandable graphite (EG)-based flame-retardant packs (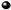: self-extinguish).

As EG had the best flame-retardant effect, EG-based packages with different mass ratios of flame retardants were chosen to further investigate. The horizontal burning results of the SSPCMs are shown in Figure 2. It can be seen that the combustion time of PCM-EG was still the longest. The PCMs with EG/RP or EG/IFR coatings had longer combustion times and were extinguished at lower loadings than those with single RP or IFR coatings. In addition, the combustion times of the SSPCMs were decreased with less EG in flame-retardant packages. Therefore, EG played a more significant role in improving the flame retardance of SSPCMs than the other flame retardants.

### 2.2. Flammability Properties

As the flame retardants increased, the samples began to self-extinguish during horizontal burning. In order to prepare the flame-retardant SSPCMs to meet the strict flammability standards, EG-based coatings with an acrylic resin/flame retardant ratio of 10/5 were adopted and their flame-retardant performances were evaluated by limiting oxygen index (LOI), vertical burning tests and cone calorimeter tests.

#### 2.2.1. Limiting Oxygen Index (LOI)

The LOI values of different SSPCMs are listed in Table 1. The LOI value of PCM was 18.7%. The LOI value of PCM-EG sharply increased to 31.8%, showing the good flame-retardant effect of the EG coating on SSPCM. Using the EG/RP and EG/IFR coatings, the LOI values were almost between the PCM and PCM-EG. More EG in the coating endowed the sample with a higher LOI value.

#### 2.2.2. Vertical Burning Test

Table 2 shows the vertical burning results of the surface-coated SSPCMs. No dripping occurred in any of the samples during burning, owing to the OMMT in the matrix that increased the melt viscosity and strength of the strips [7]. PCM burnt quickly, while PCM-EG reached the V0 flame-retardant level. The incorporation of RP or IFR into the coatings weakened the flame-retardant effects as the EG decreased and more flame retardants were needed to keep the V0 classification.

#### 2.2.3. Cone Calorimeter Test

**Figure 3:**
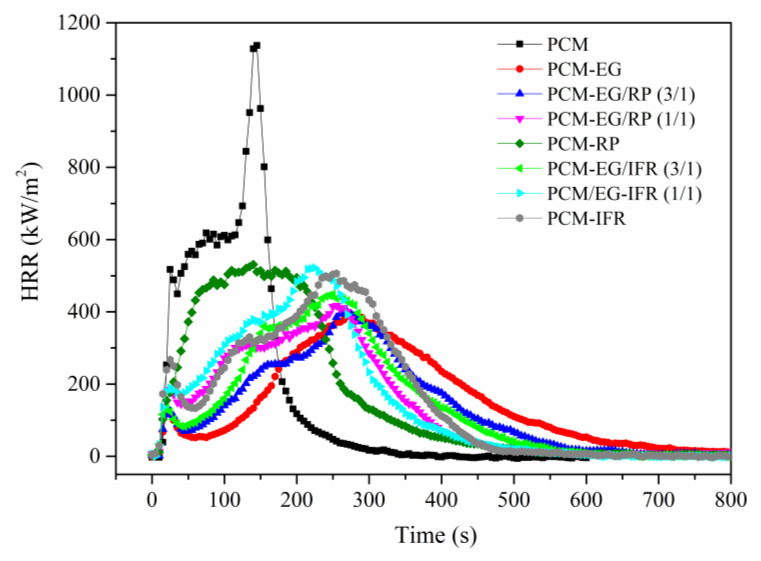
Heat release rates of SSPCMs.

The cone calorimeter test could simulate the real fire situation and evaluate the flame retardance of the materials. Figure 3 shows the heat release evolution of the different surface-coated SSPCM samples and the data obtained in the cone calorimeter tests are listed in Table 3. For PCM, the heat release rate (HRR) increased rapidly after ignition and the peak heat release rate (PHRR) reached 1137.0 kW/m^2^ in 145 s. The peak mass loss rate (PMLR) was 0.29 g/s. The total smoke production (TSP) was 18.9 m^2^. After surface treatment, the PHRRs of the samples largely decreased, the heat release processes slowed down and the PMLRs reduced. In particular, PCM-EG had the lowest PHRR of 392.5 kW/m^2^, the slowest burning process with a t_PHRR_ of 275 s and the lowest PMLR of 0.14 g/s. Moreover, PCM-EG had the smallest TSP of 14.3 m^2^. From Table 3, it was also seen that RP and IFR could promote smoke production during burning and more RP or IFR resulted in higher TSP values. In addition, the TSPs of the samples containing RP were larger than those containing IFR.

### 2.3. Flame Retardant Mechanism

To maintain a high energy storage capacity, a large proportion of paraffin is required in the SSPCM. Both polymer matrices (HDPE and SBS) and the paraffin were combustibles. As paraffin became liquid above the phase change temperature, the flame-retardant method aimed to enhance the flame-retardant performance of a material with a liquid medium, not just solid polymers.

As we know, polymers and paraffin have different combustion processes. The polymers had long molecular chains with a large molecular weight (10^4^–10^5^). During burning, they decomposed into small molecules/molecular radicals and then gasified into the air to cause continuous combustion. Paraffin had a high vapor pressure and was mainly composed of alkanes with a carbon number of 18–30. It gasified directly at a moderate temperature without decomposition and caused combustion.

**Figure 4:**
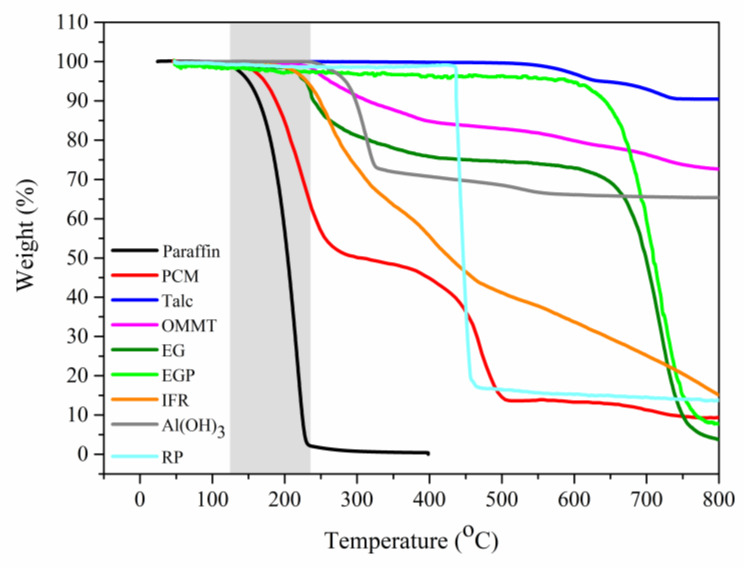
Thermogravimetric analysis (TGA) curves of paraffin, PCM and flame retardants (gray part: mass loss interval of paraffin).

This could be further clarified by the thermogravimetric analysis (TGA) curves of the paraffin and PCM shown in Figure 4. The paraffin began to evaporate before reaching 200 °C and mass loss was completed at 230 °C (see the gray part). For PCM, the first mass loss corresponded to the vaporization of paraffin and the second mass loss in the range of 400–500 °C was caused by the decomposition of SBS and HDPE. This indicated that the burning of paraffin would occur earlier and easier than the polymers. Therefore, hindering the evaporation of paraffin or improving the flame resistance of paraffin were significant for improving the flame retardancy of SSPCM substantially.

The TGA curves of the different flame retardants are presented in Figure 4. EG, RP and IFR expanded, decomposed or burnt at much higher temperatures than at which the paraffin evaporated. That is, paraffin would evaporate from the bulk to cause fires before the flame retardants acted to form protective char layers. Of course, the char layers could somewhat protect the HDPE and SBS. This could explain why the bulk addition method showed limited flame-retardant effects on SSPCM [8,9,11,12,13,16,17].

However, the surface coating strategy of putting flame retardants onto the surface of the samples showed good flame-retardant effects on the SSPCM. Especially for PCM-EG, the LOI value of 31.8% and the V0 flame-retardant level were obtained with a coating ratio of 10/5, containing 11.7 phr EG (EG content in per hundred of PCM matrix). Thus, why did the surface coating with fewer flame retardants perform much better than the bulk addition for improving the flame retardancy of SSPCM?

**Figure 5:**
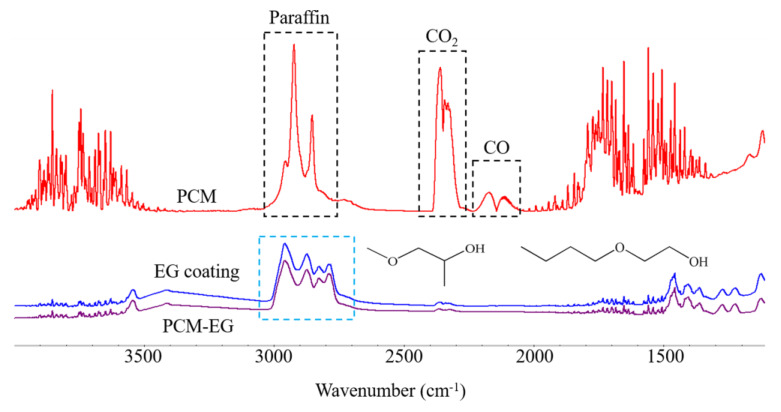
In situ Fourier transfer infrared spectrometry (FTIR) spectra of PCM, PCM-EG and EG coating at 150 °C.

Square PCM, PCM-EG and the EG coating itself (13 mm × 13 mm × 3 mm) were placed in the in situ Fourier transfer infrared spectrometry (FTIR) cell and the IR spectra of volatile components at 150 °C were detected, as shown in Figure 5. The peaks of PCM at 2923 and 2852 cm^−1^ were attributed to C-H in paraffin, indicating that the evaporation of paraffin in the PCM began even at such a low temperature. Simultaneously, peaks of CO_2_ at 2395 and 2343 cm^−1^ appeared, accompanied by the generation of CO peaks at 2173 and 2115 cm^−1^, implying the oxidation of paraffin. These results were in accordance with the TGA data analysis in Figure 4. Therefore, it is reasonable to assume that, once exposed to fire, the paraffin in PCM would quickly evaporate and start burning. After the surface coating, the peaks of PCM-EG in the area of 3000–2700 cm^−1^ were different from those of the PCM but were in accordance with the peaks of the EG coating. These peaks were attributed to the residual solvent in the coating, which was confirmed to be long-chain ether alcohols by pyrolysis gas chromatography-mass spectrometry (Py-GC-MS). Furthermore, no obvious peaks of paraffin, CO_2_ or CO appeared. This indicated that almost no paraffin evaporated from PCM-EG, even at 150 °C and there was no oxidation of paraffin. In addition, the weight losses of the PCM, PCM-EG and EG coating were 50, 18 and 20 mg, respectively, after in situ FTIR tests. The weight loss of PCM was mainly attributed to the evaporation of the paraffin. The weight losses of the PCM-EG and EG coating were similar, mainly corresponding to the residual solvent evaporation from the coating. Therefore, the coating could effectively prevent the paraffin in the PCM-EG from evaporating outside during heating.

**Figure 6:**
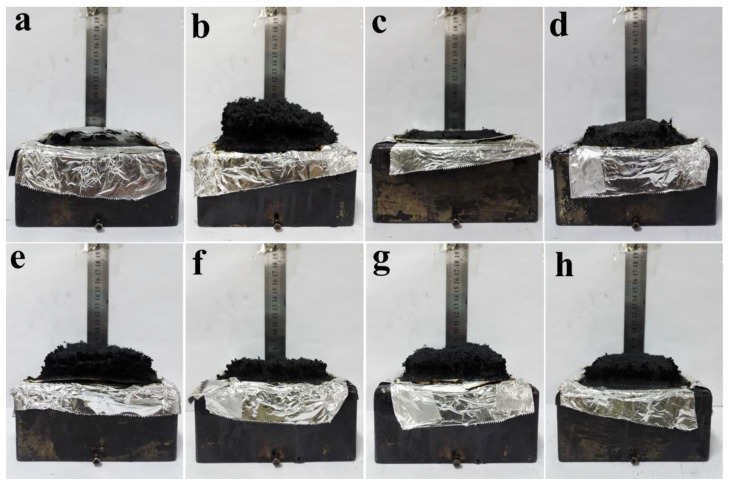
Photos of SSPCM residues after cone calorimeter tests: (**a**) PCM, (**b**) PCM-EG, (**c**) PCM-red phosphorus (RP), (**d**) PCM-intumescent flame retardant (IFR), (**e**) PCM-EG/RP (3/1), (**f**) PCM-EG/RP (1/1), (**g**) PCM-EG/IFR (3/1) and (**h**) PCM-EG/IFR (1/1).

**Figure 7:**
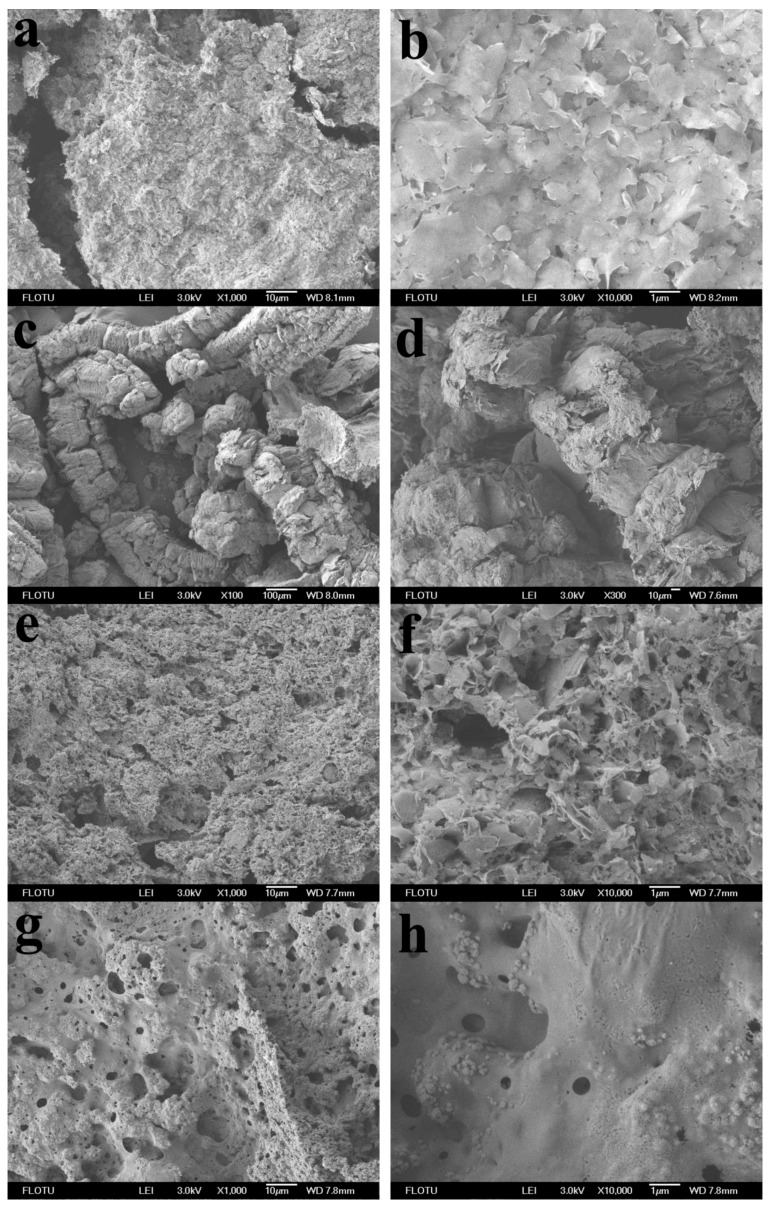
Field emission scanning electron microscopy (FESEM) micrographs of SSPCM residues after the cone calorimeter tests: (**a**,**b**) PCM, (**c**,**d**) PCM-EG, (**e**,**f**) PCM-RP and (**g**,**h**) PCM-IFR.

Photos and field emission scanning electron microscopy (FESEM) micrographs of SSPCM residues after cone calorimeter tests are shown in Figure 6 and 7, respectively. PCM-EG had the thickest carbon layer (Figure 6b), as EG expanded rapidly into a porous structure at a high temperature (Figure 7c,d). The expansion dissipated massive heat to slow down the heat transfer rate and thus delayed the gasification of paraffin from the matrix. The formed porous structure trapped lots of combustibles, such as gaseous paraffin and degradation products and retarded the burning process greatly. Therefore, the EG coating performed a good flame-retardant effect on PCM. The importance of the expansion process for the flame resistance of SSPCM could be also reflected from the TGA curve of EGP. In contrast to EG, EGP did not expand at a high temperature and only acted as a physical barrier, like Talc, so the flame-retardant effect was quite limited (see in Figure 1).

For PCM-RP, no expansion occurred (Figure 6c). Some phosphoric acid or phosphoric oxides were generated during burning and attached to the surface of the OMMT (Figure 7e,f). These products largely increased smoke and the TSP values increased, as shown in Table 3.

For PCM-IFR, a compact intumescent carbon layer was formed during burning (Figure 6d and Figure 7g,h). It could block some smoke production, hence the samples containing IFR had lower TSP values than those with RP.

**Figure 8:**
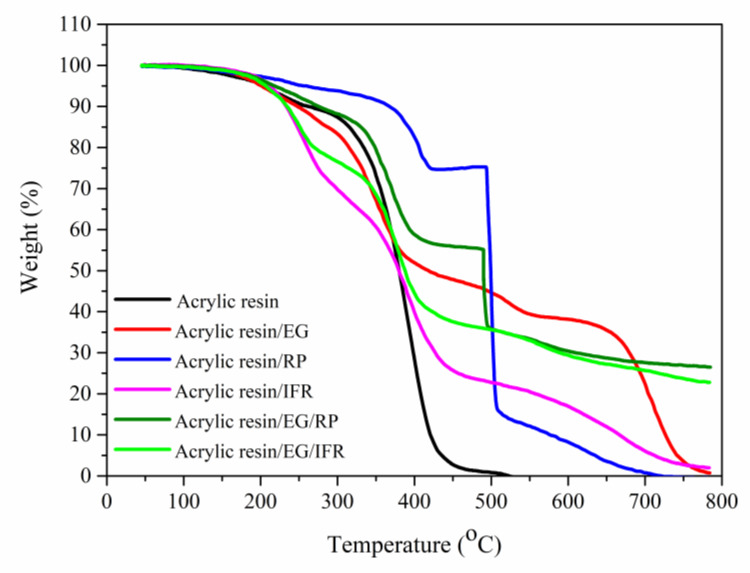
TGA curves of different flame-retardant coatings.

For PCM-EG/RP and PCM-EG/IFR, the carbon layers became thinner than PCM-EG, mainly due to the decreased EG contents. In addition, the full expansion of EG would be somewhat limited by the decomposition products of RP or the intumescent layers of IFR. As a result, the flame-retardant effects were weakened as the EG loadings decreased. However, EG/RP and EG/IFR maintained more residues than individuals at a high temperature (Figure 8), owing to synergistic protection from EG and some phosphorus oxides or intumescent carbon layers. The strengthened residues helped to keep lightweight expanded graphite from escaping during burning.

**Figure 9:**
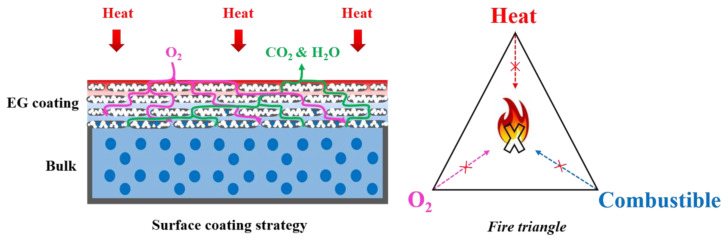
The flame-retardant mechanism of surface-coated SSPCM.

In summary, when the bulk flame retardant SSPCM is exposed to flames, the paraffin in the matrix easily vaporizes to cause combustion before the flame retardants can form a protective carbon char layer, as the reaction temperatures of common flame retardants are higher than the gasification and oxidation temperature of paraffin and they are unable to suppress the burning of gaseous paraffin. Thus, bulk flame-retardant SSPCM shows limited flame retardance. When the surface-coated SSPCM is exposed to flames, there is a new flame-retardant mechanism, as presented in Figure 9. At a low/moderate temperature, the flame retardants in the coating have yet to expand and decompose, so the flame-retardant coating acts as a physical barrier. Although a very small amount of gaseous paraffin generates, it is sealed inside the coating. As the temperature increases, the coating expands and decomposes to form a thick porous carbon char layer. The expansion is endothermic and are able to slow down the temperature rise of the sample. Thus, the temperature of the sample is relatively low, and not much paraffin evaporates and little polymer matrix decomposes as combustibles, despite the high flame temperature. Moreover, the small amount of combustibles can be trapped in the porous layer. Therefore, few combustibles are directly exposed to the flame and oxygen and the combustion is extremely suppressed. In a word, the coating blocks the transfer of combustibles, oxygen and heat between the sample and the environment. Hence, SSPCMs with a flame-retardant coating, especially EG coating, exhibit good flame-retardant properties with much fewer flame retardants and self-extinguishing can be achieved. In addition, as fewer flame retardants are placed on the surface of the SSPCM instead of the matrix, the storage capacity will not be sacrificed too much.

## 3. Material and Methods

### 3.1. Materials

High-density polyethylene (HDPE; J0, MI = 0.15, ρ = 0.942) was purchased from Beijing Eastern Petrochemical Co., Ltd. (Beijing, China). Styrene-butadiene-styrene star copolymer with an S/B ratio of 3/7 (SBS; T161B) was supplied by Dushanzi Petrochemical Co., Ltd. (Karamay, China). Paraffin, with a phase change temperature of 28 °C and latent heat of 133 J/g, was obtained from the Shijiazhuang Zhongdejieneng Phase Change Material Company (Shijiazhuang, China). Talc, organic montmorillonite (OMMT; NB901) and expandable graphite (EG; expansion ratio 100–400 mL/g) were provided by Beijing Guoli Superfine Powder Co., Ltd. (Beijing, China); Zhengjiang Huate Chemical Co., Ltd. (Hangzhou, China); and Qingdao Nanshuhongda Graphite Products Co., Ltd. (Shandong, China), respectively. Ammonium polyphosphate (APP; Preniphor TM EPFR-APP231) was supplied by Starbetter Chemical Products Co., Ltd. (Beijing, China). Pentaerythritol (PER), melamine (MA) and Al(OH)_3_ were purchased from Beijing Chemical Works (Beijing, China). Red phosphorus (RP) was provided by Tianjin Fuchen Chemical Reagents Factory (Tianjin, China). Acrylic resin (FB9788) was purchased from Shanghai Fengbiao Chemical Technology Co., Ltd. (Shanghai, China) The acetone was from Modern Oriental (Beijing) Technology Development Co., Ltd. (Beijing, China) The expanded graphite plate (EGP) was homemade from expanded graphite sheared in hexane by a homogenizer (FM200, Fluko Equipment Shanghai Co., Ltd., Shanghai, China) at 10,000 r/min for 20 min. The intumescent flame retardant (IFR) was a mixture of APP, PER and MA, with a mass ratio of 1:1:1.

### 3.2. Preparation of SSPCMs

The SBS, HDPE, paraffin and OMMT (SBS/HDPE/paraffin/OMMT = 30/10/60/20) were physically mixed and heated at 60 °C for 30 min. The mixture was then melt-blended in a torque rheometer (RM-200C, Harbin Hapro Electric Technology Co., Ltd., Harbin, China) at 140 °C for 6 min at 60 rpm (termed PCM). The PCM was hot-pressed into strips (130 mm × 13 mm × 3 mm, for horizontal burning, vertical burning and limiting the oxygen index tests) or plates (13 mm × 13 mm × 3 mm, for in situ FTIR measurement, and 100 mm × 100 mm × 3 mm, for the cone calorimeter tests) under a pressure of 8 MPa for 4 min at 140 °C.

### 3.3. Preparation of Surface-Coated SSPCMs

A flame-retardant coating was prepared by dispersing a flame retardant into the acrylic resin solution in acetone. The mass ratios of acrylic resin to flame retardant were 10/1–10. The compositions of the surface-coated SSPCMs are listed in Table 4. The samples were brushed with coatings and dried naturally to remove acetone in a fume hood at room temperature (termed PCM-A, with A referring to the flame retardant). The final thickness of the coatings was ~300 μm.

### 3.4. Characterization

#### 3.4.1. Horizontal Burning Test

The sample strips (130 mm × 13 mm × 3 mm) were clamped horizontally with tongs at one end. They were ignited at the other end for 10 s. The burning time until the flames spread to the tongs was recorded.

#### 3.4.2. Vertical Burning Test

The sample strips were clamped vertically over cotton wool and ignited twice. Each ignition was for 10 s. A best V0 level was achieved when the total burning time was less than 10 s and no dripping was generated, whereas a poorest not classified (NC) level was marked if the total burning time exceeded 30 s or the flames spread to the tongs.

#### 3.4.3. Limiting Oxygen Index (LOI) Test

The limiting oxygen index tests of the sample strips were carried out using a Dynisco LOI instrument (Fire TestingTechnology Ltd., West Sussex, UK). The minimum concentration of oxygen, which could just support the burning of the strips, was obtained as the LOI. The higher the LOI, the better the flame-retardant property.

#### 3.4.4. Cone Calorimeter Test

The heat release rates (HRRs) of the samples were measured with a FTT0082 cone calorimeter (Fire TestingTechnology Ltd., West Sussex, UK) under a heat flux of 50 kW/m^2^, according to ISO 5660–1. The data were reported to assess the flame retardancy of the SSPCMs.

#### 3.4.5. Field Emission Scanning Electron Microscopy (FESEM)

FESEM was performed on a JEOL model JSM-7401 (JEOL Ltd., Tokyo, Japan) apparatus with an operating voltage of 3.0 kV, in order to investigate the morphology of the sample residues after the cone calorimeter tests.

#### 3.4.6. Thermogravimetric Analysis (TGA)

TGA (TGA50, Shimadzu, Japan) was used to detect the weight loss of a sample during the heating process and to evaluate the thermal stability of the sample. The test temperature ranged from 50 to 800 °C at a heating rate of 10 °C/min, under an air gas flow of 25 mL/min.

#### 3.4.7. In situ Fourier Transfer Infrared Spectrometry (FTIR)

**Figure 10:**
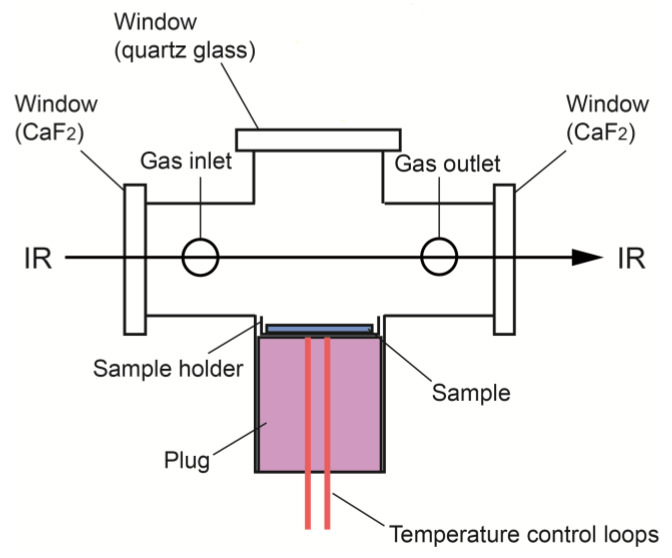
Schematic diagram of the sealable cell [23]. Copyright Elsevier, 2016.

An in situ FTIR accessory that was developed in our laboratory [23] was applied to analyze the volatile components from the SSPCM. A schematic diagram is given in Figure 10. Square samples (13 mm × 13 mm × 3 mm) were placed in the FTIR cell and the gas inlet and outlet were closed. The cell was put into the sample compartment of the FTIR spectrometer (Thermo-Nicolet iS10, Thermo Fisher Scientific, Massachusetts, USA). The sample was heated stepwise to 150 °C, volatile components evaporated from the sample into the cell and the in situ FTIR signals of the cell atmosphere were collected.

#### 3.4.8. Pyrolysis Gas Chromatography-Mass Spectrometry (Py-GC-MS)

An EGA/PY-3030D multi-shot pyrolyzer (Frontier Laboratories Ltd., Fukushima, Japan) was installed on a gas chromatography-mass spectrometry instrument (GCMS-QP2010 SE, Shimadzu, Japan). The flash evaporation technique was used to separate the volatile components from the samples by heating them at 150 °C for 0.5 min. The volatiles were then carried by He carrier gas through an Ultra ALLOY-5 column (5% diphenyl and 95% dimethylpolysiloxane, 30.0 m, i.d. 0.25 mm × 0.05 μm stationary phase thickness) and the mixture was separated. The oven temperature was held at 50 °C for 1 min, then raised at a rate of 8 °C/min to 300 °C and held for 30 min. Finally, each component was identified by an MS detector with an EI source and a mass range of *m/z* 33–600. The MS spectrum was compared to the standards in the NIST 11 database, composed of ca. 240,000 standard spectra.

## 4. Conclusions

The paraffin in the SSPCM easily evaporates from the matrix below 230 °C because of its high vapor pressure. Hence, it contributes to the higher production of combustibles and causes combustion before any flame retardants decompose to protect the matrix. Thus, the bulk addition method showed a limited flame-retardant effect on the SSPCM, even with 20–40 wt% of flame retardants in the matrix. After being surface coated, PCM-EG exhibited the best flame retardance. The horizontal burning time was the longest at low loadings of EG, the LOI value was up to 30%, and the V0 flame-retardant level was achieved. In addition, the peak heat release rate was declined from 1137.0 to 392.5 kW/m^2^ and the combustion process was prolonged remarkably. As the EG content in the EG-based coating decreased, the flame-retardant effect was weakened. The EG-based coating was able to suppress the volatilization of paraffin in the matrix at a moderate temperature. When the temperature kept increasing, the EG-based coating expanded and formed thick porous carbon char layers. The expansion dissipated a lot of heat and prevented the temperature of the matrix from increasing rapidly, which helped to delay the gasification of the paraffin. In addition, the thick porous char layers absorbed combustibles and hindered the diffusion of oxygen and heat inside. Therefore, the surface coating enhanced the flame retardance of SSPCM efficiently. This work will be helpful to prepare flame-retardant SSPCMs and to provide a possible solution for improving the flame-retardant property of a material containing a liquid medium in the matrix as well.

## Figures and Tables

**Table 1:** Limiting oxygen index (LOI) values of different SSPCMs (acrylic resin/flame retardant = 10/5).

Sample	LOI/%
PCM	18.7
PCM-RP	23.6
PCM-IFR	22.8
PCM-EG	31.8
PCM-EG/RP (3/1)	32.2
PCM-EG/RP (1/1)	26.8
PCM-EG/IFR (3/1)	29.8
PCM-EG/IFR (1/1)	24.2

**Table 2:** Vertical burning results of different SSPCMs (acrylic resin/flame retardant = 10/5).

Sample	Dripping	Classification
PCM	No	NC
PCM-EG	No	V0
PCM-EG/RP (3/1)	No	V0
PCM-EG/RP (1/1)	No	V0
PCM-EG/RP (1/3)	No	V1
PCM-RP	No	NC
PCM-EG/IFR (3/1)	No	V0
PCM-EG/IFR (1/1)	No	V1
PCM-EG/IFR (1/3)	No	NC
PCM-IFR	No	NC

**Table 3:** Data from the cone calorimeter tests for SSPCMs (acrylic resin/flame retardant = 10/5).

Sample	PHRR (kW/m^2^)	t_PHRR_ (s)	THR (MJ/m^2^)	PMLR (g/s)	Mass Loss (wt%)	PEHC (MJ/kg)	Mean EHC (MJ/kg)	TSP (m^2^)
PCM	1137.0	145	109.3	0.29	90.9	79.5	39.6	18.9
PCM-EG	392.5	275	122.0	0.14	81.0	79.9	37.7	14.3
PCM-EG/RP (3/1)	399.3	265	107.1	0.18	82.6	77.4	31.7	25.2
PCM-EG/RP (1/1)	417.0	255	109.9	0.19	81.7	75.8	33.1	32.3
PCM-RP	531.2	140	126.4	0.25	84.8	77.5	34.5	39.6
PCM-EG/IFR (3/1)	448.0	250	114.2	0.20	81.8	77.8	35.5	20.9
PCM-EG/IFR (1/1)	522.3	225	118.8	0.19	84.1	78.5	35.0	23.5
PCM-IFR	506.5	255	126.8	0.21	89.0	74.5	33.3	25.3

PHRR—peak heat release rate; THR—total heat release; PMLR—peak mass loss rate; PEHC—peak effective heat combustion; TSP—total smoke production.

**Table 4:** Compositions of surface-coated SSPCMs.

Sample	Flame Retardant
PCM	None
PCM-Talc	Talc
PCM-OMMT	OMMT
PCM-EG	EG
PCM-EGP	EGP
PCM-APP	APP
PCM-IFR	IFR
PCM-Al(OH)_3_	Al(OH)_3_
PCM-RP	RP
PCM-EG/RP (3/1)	EG/RP = 3/1
PCM-EG/RP (1/1)	EG/RP = 1/1
PCM-EG/RP (1/3)	EG/RP = 1/3
PCM-EG/IFR (3/1)	EG/IFR = 3/1
PCM-EG/IFR (1/1)	EG/IFR = 1/1
PCM-EG/IFR (1/3)	EG/IFR = 1/3
